# Automated inversion time selection for black-blood late gadolinium enhancement cardiac imaging in clinical practice

**DOI:** 10.1007/s10334-023-01101-2

**Published:** 2023-06-09

**Authors:** Aurélien Maillot, Soumaya Sridi, Xavier Pineau, Amandine André-Billeau, Stéphanie Hosteins, Jean-David Maes, Géraldine Montier, Marta Nuñez-Garcia, Bruno Quesson, Maxime Sermesant, Hubert Cochet, Matthias Stuber, Aurélien Bustin

**Affiliations:** 1 IHU LIRYC, Electrophysiology and Heart Modelling Institute, Université de Bordeaux, INSERM, U1045, Avenue du Haut-Lévêque, 33604 Pessac, France; 2 Department of Cardiovascular Imaging, Hôpital Cardiologique du Haut-Lévêque, CHU de Bordeaux, Avenue de Magellan, 33604 Pessac, France; 3 Department of Diagnostic and Interventional Radiology, Lausanne University Hospital and University of Lausanne, Lausanne, Switzerland; 4 INRIA, Université Côte d’Azur, Sophia Antipolis, Nice, France; 5 CIBM Center for Biomedical Imaging, Lausanne, Switzerland

**Keywords:** Myocardial infarction, Gadolinium Enhancement, Magnetic resonance imaging, Black-blood imaging

## Abstract

**Objective:**

To simplify black-blood late gadolinium enhancement (BL-LGE) cardiac imaging in clinical practice using an image-based algorithm for automated inversion time (TI) selection.

**Materials and methods:**

The algorithm selects from BL-LGE TI scout images, the TI corresponding to the image with the highest number of sub-threshold pixels within a region of interest (ROI) encompassing the blood-pool and myocardium. The threshold value corresponds to the most recurrent pixel intensity of all scout images within the ROI. ROI dimensions were optimized in 40 patients’ scans. The algorithm was validated retrospectively (80 patients) versus two experts and tested prospectively (5 patients) on a 1.5 T clinical scanner.

**Results:**

Automated TI selection took ~ 40 ms per dataset (manual: ~ 17 s). Fleiss’ kappa coefficient for automated-manual, intra-observer and inter-observer agreements were $\overline{\kappa }$= 0.73, $\overline{\kappa }$ = 0.70 and $\overline{\kappa }$ = 0.63, respectively. The agreement between the algorithm and any expert was better than the agreement between the two experts or between two selections of one expert.

**Discussion:**

Thanks to its good performance and simplicity of implementation, the proposed algorithm is a good candidate for automated BL-LGE imaging in clinical practice.

**Supplementary Information:**

The online version contains supplementary material available at 10.1007/s10334-023-01101-2.

## Introduction

Bright-Blood Late Gadolinium Enhancement (BR-LGE) cardiovascular magnetic resonance (CMR) imaging is the reference technique for the assessment of regional scar formation and myocardial fibrosis [1–3]. In BR-LGE imaging, nulling of the viable myocardial signal using inversion recovery pulses enables scar visualization by providing a high contrast between healthy and injured myocardium after contrast injection. However, for myocardial scars adjacent to the blood chambers, the presence of high signal intensity from the blood pool often hinders their accurate visualization and delineation, particularly for subendocardial scars [4]. To circumvent this problem, Black-Blood LGE (BL-LGE) imaging techniques have been proposed to null both healthy myocardium and blood pool signals and to provide high scar-to-blood and high scar-to-viable myocardium contrast [4–8]. BL-LGE techniques are increasingly being used in clinical practice thanks to their unique scar visualization capabilities [9, 10]. It has been recently shown that BL-LGE imaging could ascertain or rule out a diagnosis otherwise inconclusive on BR-LGE imaging in a significant number of patients [11]. Optimal contrast, however, depends on the selection of optimal sequence timing parameters that need to be tailored patient-wise before the acquisition. This step is of utmost importance as it directly impacts the scar visualization and delineation.

In clinical routine, timing parameter selection is performed by MR operators by selecting the optimal times from a series of 2D short-axis images acquired with different timing parameters. This manual process increases the complexity of the BL-LGE acquisition while adding to the workload of the MR operator. Moreover, like with any manual process, it can be prone to both inter- and intra-observer variability. Automation of timing parameter selection could therefore be beneficial to accelerate and standardize the LGE workflow, increase the exam reproducibility, while reducing the MR operator workload. To the best of our knowledge, no algorithms have been proposed for automated BL-LGE parameters selection.

Automation of contrast selection is a small, yet necessary, incremental improvement toward a more accurate, effortless, and time efficient procedure for myocardial scar characterization. We propose an image-based algorithm for automated contrast selection in magnetization-prepared BL-LGE [11]. This BL-LGE sequence has the advantage of having only one parameter to scout: the Inversion Time (TI), which is the delay between the preparation pulse and the data acquisition. We purposefully develop an automated algorithm based on simple image-processing techniques to facilitate its integration to MRI scanners and thus promote rapid clinical integration. The performance of the proposed algorithm was first assessed retrospectively in 120 patients and its integration in clinical routine was then tested prospectively in 5 patients with known or suspected structural heart disease.

## Materials and methods

Optimal TI selection was performed by detecting the scout image with the highest number of low-intensity pixels within a ROI located inside the heart and containing parts of the ventricular blood pool and myocardium. The algorithm was implemented in C+ + without external libraries to enable its seamless incorporation to any MRI scanner. A retrospective study was conducted in 120 patients and a prospective study was conducted in 5 patients. All patients underwent CMR for known or suspected structural heart disease. The study was approved by the Biomedical Research Ethics Committee and all participants provided informed consent for participation.

### Data acquisition

Acquisitions were performed on a 1.5 T clinical scanner (MAGNETOM Aera, Siemens Healthcare, Erlangen, Germany), using an 18-element body coil and a 32-element spine coil. A prototype electrocardiogram-triggered 2D single-shot, balanced steady-state free-precession BL-LGE scout was performed prior to a fast free-breathing motion-compensated T1ρ-prepared BL-LGE sequence [11]. A schematic overview of the sequence is depicted in Figure Online Resource 1 and acquisition parameters are listed in Table 1. The BL-LGE sequence was performed at the end of the CMR exam, about 12–15 min after injection of 0.2 mmol/kg gadoteric acid. The TI scout sequence was run before BL-LGE imaging and consisted in 11 mid-ventricular single-shot short-axis 2D images acquired during free-breathing with TIs ranging from 60 to 160 ms with a 10 ms increment. The preparation duration (27 ms) was optimized beforehand with an extended phase graph simulation [11] so that the TI range provides at least one image of the scout with black blood and black healthy myocardium at about 12–15 min post injection.

**Table 1:** Acquisition parameters and patient demographics for the offline retrospective and inline prospective automated BL-LGE studies

Acquisition parameters		
Sequence	TI scout	BL-LGE
Acquisition	2D T1ρ-prepared	2D T1ρ-prepared bSSFP
Magnetic field (Tesla)	1.5	1.5
Coverage	Mid-ventricular	Whole-heart
Repetition time (ms)	2.9	2.9
Echo time (ms)	1.2	1.2
Flip angle (degree)	60	60
Field of view (mm)	380 × 315	380 × 315
Acquired resolution (mm)	2.0 × 1.5 × 6.0	2.0 × 1.5 × 6.0
Reconstructed resolution (mm)	1.5 × 1.5 × 6.0	1.5 × 1.5 × 6.0
Number of slices (median [Q1–Q3])	1	14 [14–16]
Phase oversampling (%)	0	0
Slice oversampling	No	No
Asymmetric echo	Yes	Yes
Acquisition window (ms)	160	160
T1ρ duration (ms)	27	27
T1ρ frequency (Hz)	500	500
Scan acceleration	GRAPPA × 2	GRAPPA × 2
Trigger pulse (RR interval)	2	2
Bandwidth (Hz/pixels)	849	849
Free-breathing	Yes	Yes
Motion compensation	No	Yes
Inversion time range (ms)	60–160	n/a
Inversion time increment (ms)	10	n/a
Images per scout	11	n/a

Patients’ characteristics		
	Offline study	Inline study
Number of patients	120	5
Gender (F/M)	15/85	2/3
Age [range] (years)	[17–83]	[39–74]

*bSSFP* balanced steady-state free-precession, *RR* time between two R waves, *GRAPPA* generalized autocalibrating partially parallel acquisitions

### Automated image-based TI algorithm

**Fig. 1:**
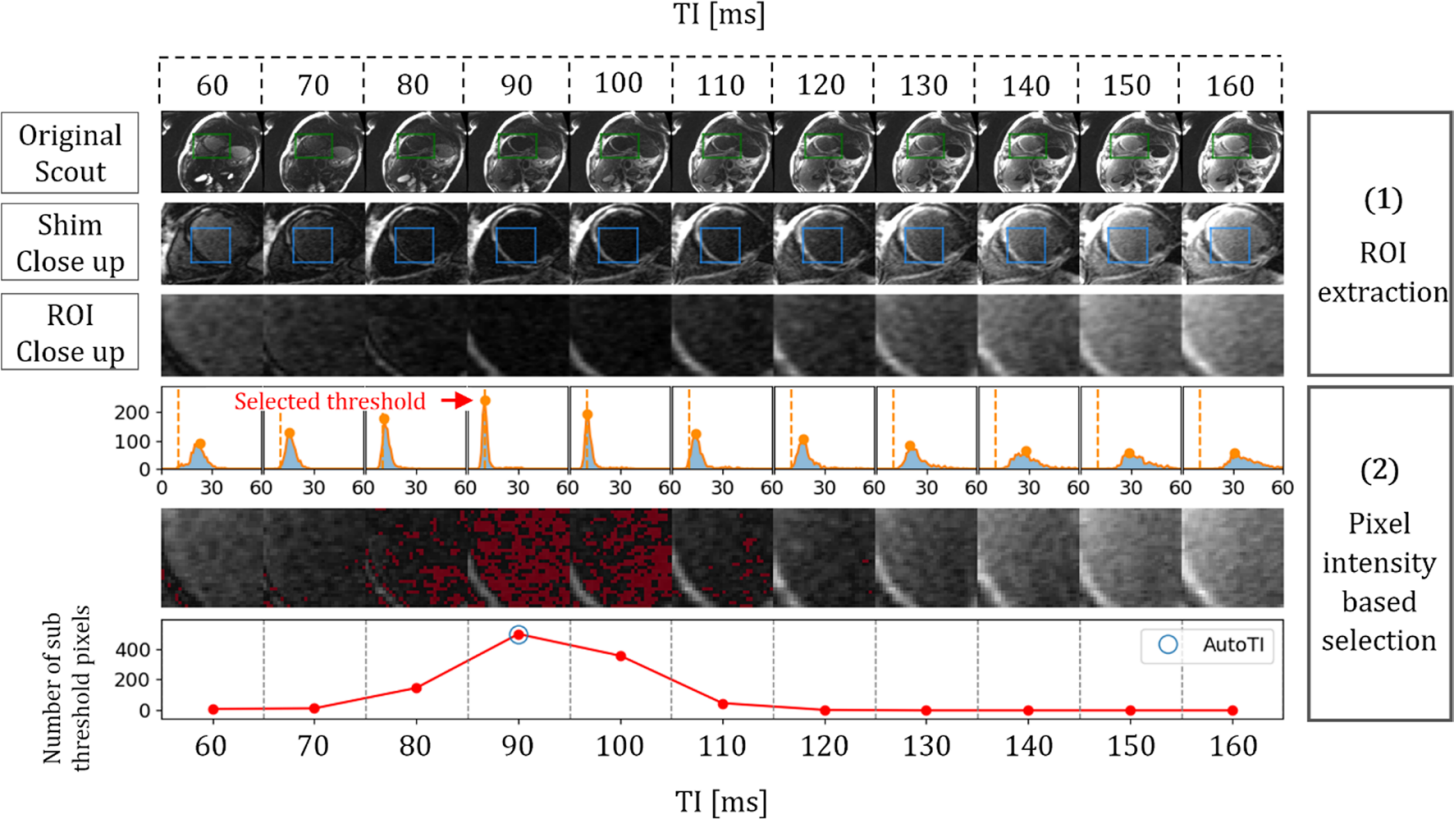
Automated TI selection process through one patient’s example. (1) ROI extraction: First row: coarse detection of the heart on the original scout images using the 2D shim box information (green rectangle). Second row: extraction of a Region of Interest (ROI) containing information from the myocardium and the blood pool (blue rectangle). (2) Pixel Intensity based selection: one dimensional histograms are computed for the pixels within the ROI. Maximums of the histograms are detected (orange circles) and the intensity value (dashed orange line) corresponding to the global maximum over all images (red arrow) is selected for thresholding. Sub-threshold pixels are displayed in red and the TI corresponding to the image with the highest number of sub-threshold pixels is selected (blue circle)

For the sake of simplicity and explainability, the proposed algorithm purposefully mimics the manual selection process which consists of selecting, from the series of scout images, the image with the highest scar-blood and scar-viable myocardium contrast. In practice, this image has the lowest signal intensity within the ventricular blood pool and the healthy myocardium. To do so, the automated algorithm operates on the TI scout images in two distinct steps. Step 1: Extraction of a region of interest (ROI), containing relevant information about the ventricular blood pool and myocardium. Step 2: Selection of the image with the highest number of low intensity pixels within this ROI. The corresponding TI is then used for the subsequent whole-heart BL-LGE acquisition. The overall process is depicted in Fig. 1 while the different steps are detailed below.

#### Step 1: ROI extraction

Accurate localization of the myocardium and ventricular blood pools would lead to optimal ROI selection. However, because our T1-rho pulse has been thoroughly optimized using extended phase graph simulation to cancel out blood and healthy myocardium signals at the same TI, the optimization can be performed equally well on a unique ROI rather than on two individual ones. Therefore, this problem can be greatly simplified by selecting a coarse ROI that includes pixels from the blood pools only or from both the blood pools and the myocardium.

ROI extraction is performed in two steps: (A) Coarse detection of the heart within the image and (B) Selection of a ROI within the heart. Acquisition of 2D CMR scout images requires the positioning of a rectangular shim box centered on the heart. This information can be exploited to easily obtain the coarse detection of the heart within the image (Online Resource 2).

Optimization of the shape and dimension of the ROI within the heart is detailed in the *offline retrospective study* section.

#### Step 2: optimal TI selection

The selection is performed by detecting the image with the highest number of low-intensity pixels within the extracted ROI. To do so, for each scout image, intensity histograms of bin size 1 are calculated on the pixels within the ROI. Histogram peaks are detected and the intensity value corresponding to the global maximum over all images is selected as a common threshold value $\left({S}_{\mathrm{thr}}\right)$. The global maximum can be used as threshold because well-attenuated signals exhibit a narrower range of pixel intensities compared to less well-attenuated signals. The image with the highest number of pixels under this threshold is defined as the image with the best signal nulling and its associated TI is selected as optimal TI. The overall process is mathematically expressed in Eq. 1 where *N* corresponds to the number of TI scout images, ${h}_{n}(i)$ is the number of occurrences of gray level *i* of a given image *n*, max PI is the maximum pixel intensity, $g\left(n\right)$ is the number of sub-threshold pixels of a given image *n,* and *α* is the index of the image with the highest histogram’s peak:

1 $${\text{TI}}_{{{\text{auto}}}} = \mathop {\arg \max }\limits_{n = 1, \ldots , N} g\left( n \right)$$

2 $$g\left( n \right) = \mathop \sum \limits_{i = 0}^{{\max {\text{PI}}}} \left( {h_{n} \left( i \right) < S_{{{\text{thr}}}} } \right)$$

3 $$S_{{{\text{thr}}}} = \mathop {\arg \max }\limits_{{i = 0, \ldots ,\max {\text{PI}}}} \left( {h_{\alpha } \left( i \right)} \right)$$

4 $$\alpha = \mathop {\arg \max }\limits_{n = 1, \ldots ,N} \left( {\mathop {\max }\limits_{{i = 0, \ldots ,\max {\text{PI}}}} h_{n} \left( i \right)} \right)$$

### Offline retrospective study

The proposed algorithm was first implemented offline and tested on TI scouts retrospectively collected on 120 patients (Table 1). It was compared with manual annotations performed by an experienced and a junior CMR operator (13 and 3 years’ experience in CMR). 40 scout datasets were used for parameters optimization and 80 for validation. Manual annotations were performed on a dedicated Graphical User Interface providing the same functionalities (scrolling, zooming, contrast and magnification adjustments) as the PACS system. The shim boxes were positioned manually by the MR operators. To ensure a reproducible positioning across different patients and exams, the simple guideline of placing the shim box outlines in direct contact with the border of the myocardium was given. Annotations were performed twice by each expert, with one week delay interval, to assess inter- and intra-observer variability.

#### Optimization

The shape and dimensions of the ROI were optimized offline on 40 patients. Rectangular and circular shapes were considered with 31 dimensions ranging from the size of the shim box $( \frac{{\mathrm{FOV}}_{\mathrm{shim}}}{1} )$ to one-fourth of its size $( \frac{{\mathrm{FOV}}_{\mathrm{shim}}}{4} )$ with a decrement factor of 0.1. The best parameters were used for the subsequent validation study.

#### Validation

Inter-observer, intra-observer and automated-manual agreements in optimal TI selection were assessed on 80 patients using Fleiss’ kappa coefficient. Because kappa analysis only provides a measure of complete agreement and does not consider the extent of differences, partial agreements were also investigated by computing the mean absolute differences and the percentage of matched optimal TIs between selections. Acquisitions with differences higher than one image (TI difference > 10 ms) were visually inspected for qualitative assessment. The time for manual TI selection was recorded for one expert.

### Inline prospective study

The automated BL-LGE sequence was integrated on a 1.5 T clinical scanner (Siemens MAGNETOM Aera, Erlangen, Germany) and was tested prospectively on 5 patients with known or suspected structural heart disease (Table 1). The updated sequence incorporates a specialized function for automated TI selection, positioned immediately after the TI scout and prior to the BL-LGE acquisition. This automated TI function takes in the TI scout images as input, conducts the necessary calculations across all images, and then outputs the optimal TI value. The optimal TI is displayed alongside its corresponding scout images on the Siemens’s interface, and subsequently utilized for the entirety of the whole-heart BL-LGE acquisition process. Inline algorithm processing time was recorded. A head-to-head comparison between BL-LGE acquisition with manual and automated TI selection was not conducted due to the need for a double administration of the contrast agent with a sufficient delay to ensure complete washout of the initial injection.

## Results

### Offline retrospective study

#### Optimization

Figure Online Resource 3 shows the ROI parameters’ optimization on 40 patients. Rectangular ROI of size $\frac{{\mathrm{FOV}}_{\mathrm{shim}}}{2.5}$ and circular ROI of size $\frac{{\mathrm{FOV}}_{\mathrm{shim}}}{2.2}$ led to the same minimum absolute mean TI difference with manual expert (2.25 ± 4.18 ms). The variation in algorithm performance was less than 1.2 ms when using ROI around these minimums, with sizes ranging between FOV_shim_/1.5 and FOV_shim_/3.0. The algorithm, with a rectangular ROI of size $\frac{{\mathrm{FOV}}_{\mathrm{shim}}}{2.5}$, selected the same TI than the expert in 31 out of 40 scouts (77.5%), and TI at plus or minus 10 ms (1 image) in 9 scouts (22.5%). 10 ms was the largest difference observed between the automated algorithm and the expert. Rectangular ROIs of size $\frac{{\mathrm{FOV}}_{\mathrm{shim}}}{2.5}$ were used for validation.

#### Validation

**Fig. 2:**
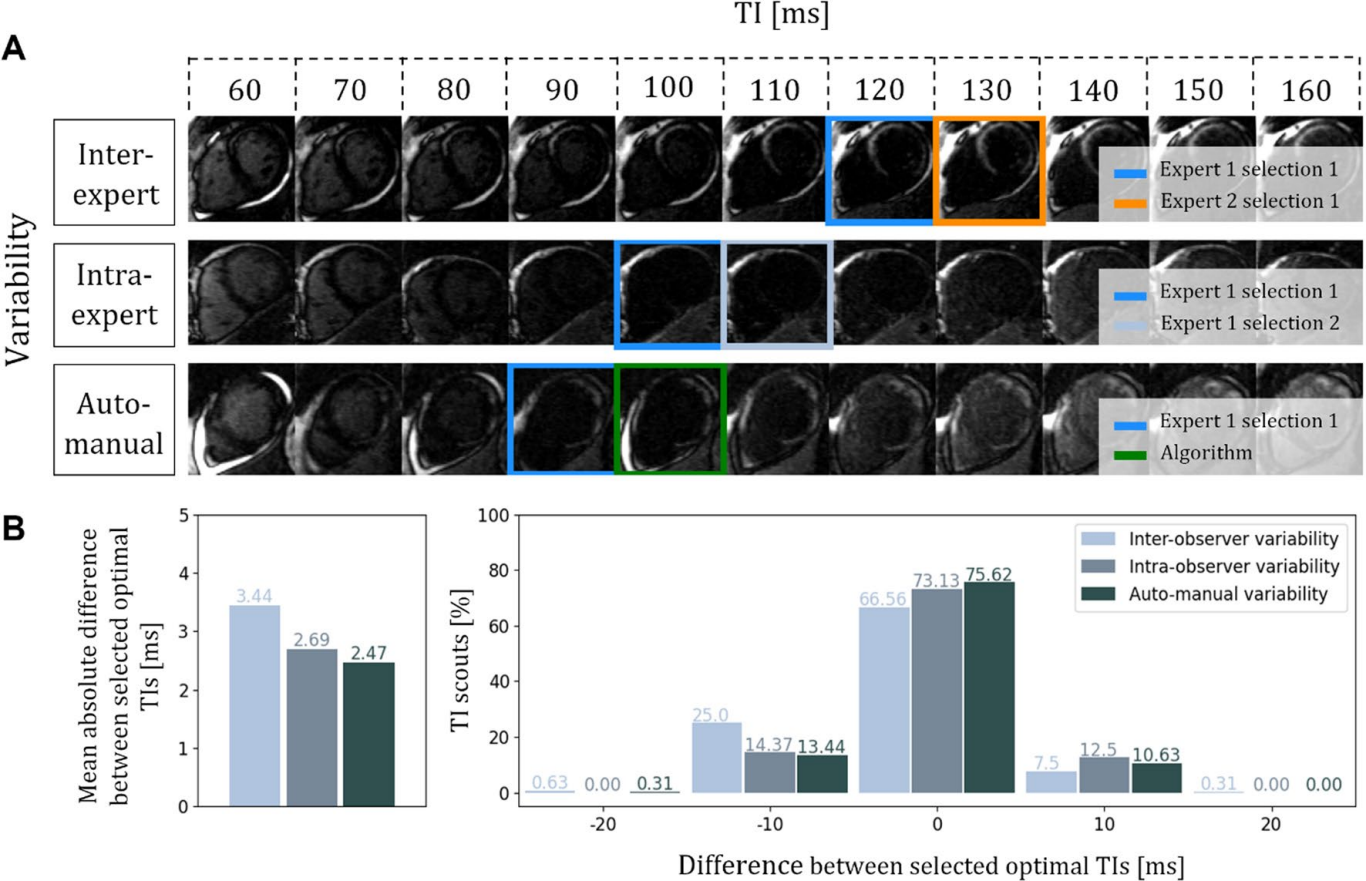
Validation of the algorithm against inter- and intra-observer variability on 80 scouts using a rectangular ROI of size $\frac{{\mathrm{FOV}}_{\mathrm{shim}}}{2.5}$. **A** Examples of inter-observer; intra-observer and auto-manual variabilities. **B** Left: mean absolute differences in TI selection. Right: percentage of matched scouts

**Fig. 3:**
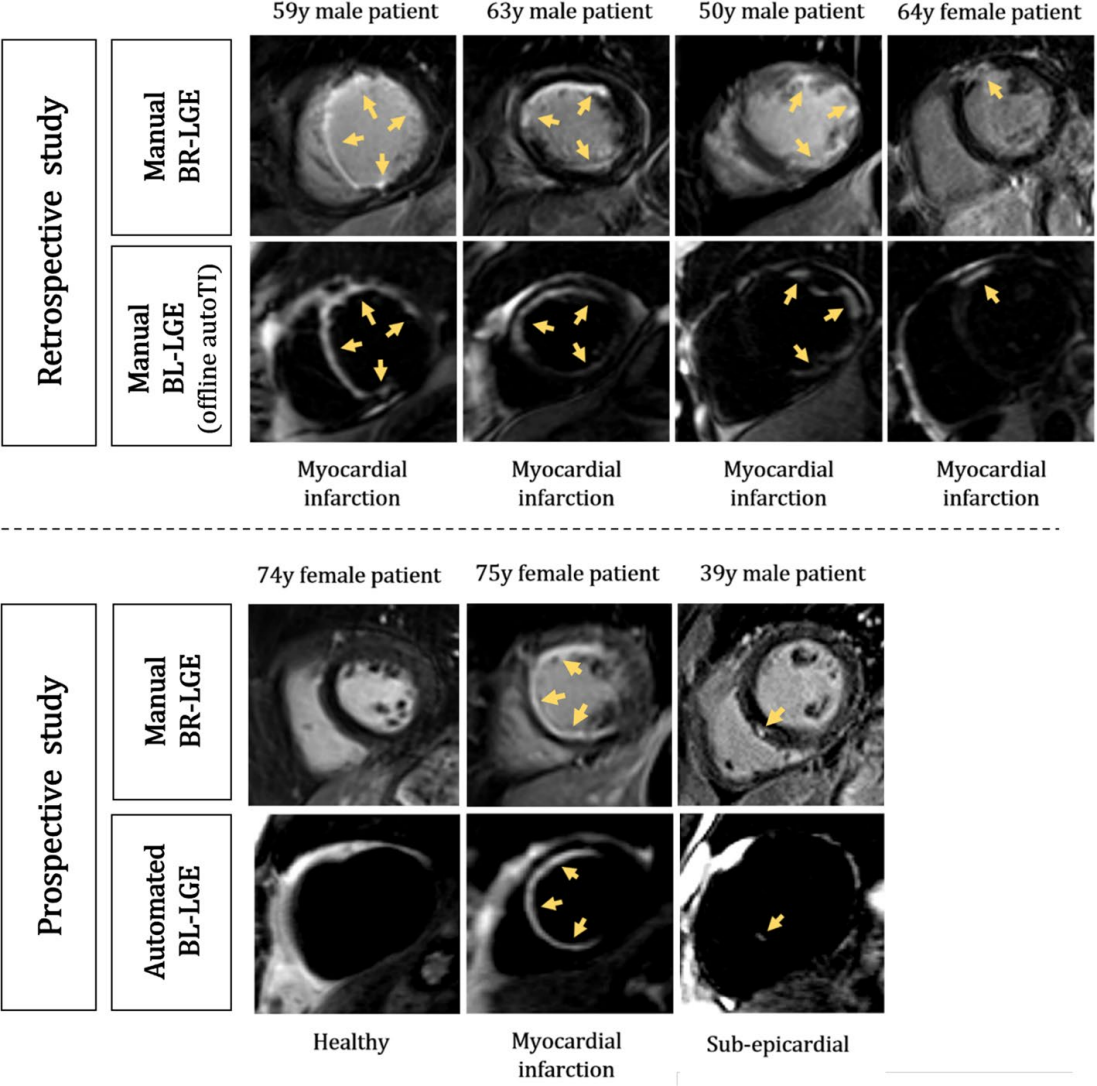
Examples of black-blood LGE images for seven different patients. Top row: four patients from the retrospective study acquired with TIs corresponding to the TIs selected by the proposed automated algorithm. Bottom row: three patients of the prospective study acquired in line with the automated BL-LGE containing the automated TI selection algorithm. Bright-blood LGE (BR-LGE) images are shown for comparison. Yellow arrows indicate areas with LGE

Table 2 and Table Online Resource 4 summarize the agreement in optimal TI selection, between the observers and the algorithm. Manual TI selection took 17.5 s ± 8.5 s. The overall inter-observer Fleiss’ kappa agreement ($\overline{\kappa }$ = 0.63) and the two experts intra-observer Fleiss’ kappa agreement (*κ* = 0.70 and 0.71) were good. The overall automated-manual Fleiss’ kappa agreement ($\overline{\kappa }$ = 0.73) was also good and similar when compared to the two experts (algorithm-expert 1, $\overline{\kappa }$ = 0.74 and algorithm-expert 2, $\overline{\kappa }$ = 0.73). The mean absolute difference in TI selection for inter-observer, intra-observer and automated-manual variability were 3.44 ± 4.94 ms, 2.69 ± 4.43 ms and 2.47 ± 4.38 ms, respectively (Fig. 2B Left). The difference between the proposed automated algorithm and any expert was therefore lower than the difference between the two experts or between one expert performing the selection twice. The same TI was selected in 75.62% of the scouts when comparing the automated algorithm to an expert, in 73.13% of the scout between two selections of the same expert and in 66.56% of the case between the two experts (Fig. 2B Right). A good agreement was found between all the selections of optimal TIs, with the highest TI difference at ± 20 ms (two images) observed only for two scouts (Figure Online Resource 5). For the first case, no single TI cancelled the signal from the healthy myocardium and blood pool while for the second case, intensity differences between the images acquired with TIs of 80, 90 and 100 ms were subtle, making human consensus difficult. BL-LGE images reconstructed with TIs corresponding to the optimal TI selected by the algorithm provided good scar visualizations (Fig. 3, top row).

**Table 2:** Fleiss kappa coefficient for optimal TI selection agreement

	Expert 1		Expert 2	
	Selection 1	Selection 2	Selection 1	Selection 2
Automated algorithm	0.68	0.79	0.78	0.67
Expert 1				
Selection 1	1	0.70*	0.62^†^	0.59^†^
Selection 2	–	1	0.70^†^	0.63^†^
Expert 2				
Selection 1	–	–	1	0.71*
Selection 2	–	–	–	1

The agreements between all selection were good with the highest overall agreement obtained between the automated algorithm and the experts ($\overline{\kappa }=$ 0.73), followed by the overall intra-expert* agreement ($\overline{\kappa }=$ 0.705) and finally by the overall inter-expert^†^ agreement ($\overline{\kappa }=$ 0.63). *κ* Strength: 0.0 ≤ * κ*  ≤ 0.2 Poor; 0.2 < * κ*  ≤ 0.4 Fair; 0.4 < * κ*  ≤ 0.6 Moderate; 0.6 < * κ*  ≤ 0.8 Good; 0.8 < * κ*  ≤ 1.0 Excellent

*Intra-expert; ^†^Inter-expert

### Inline prospective study

The algorithm took on average 40 ms on a clinical scanner to select the optimal TI from 11 images. It was 450 times faster than the manual selection process used in the previous retrospective study. The selected optimal TIs were ideal for the 5 patients and led to high quality automated BL-LGE acquisitions. The automated BL-LGE revealed the absence of scars in 3 patients and its presence in 2. Examples of single slice images extracted from inline automated BL-LGE for 3 patients are visible in Fig. 3 (bottom row). A large myocardial infarction was observed in a 75 yo female and a focal sub-epicardial scar was detected in a 39 yo male patient.

## Discussion

Optimal parameter selection is a critical step in BL-LGE imaging because it determines the contrast between fibrotic tissue, healthy myocardium, and blood-pool, which impacts the detection and delineation of scars and ultimately affects the diagnostic and prognostic value of the exam. TI selection automation would accelerate and standardize the LGE workflow, increase the exam’s reproducibility, and reduce the MR operator workload and dependency. Automation should be accurate, fast, straightforward, and explainable to facilitate usage confidence and clinical acceptance.

Because simple image processing tools cannot be exploited to automatically find the TI of BR-LGE sequences, more advanced AI-based techniques have been proposed [12, 13]. Bahrami et al. [12] proposed the use of a deep learning network using spatial and temporal imaging characteristics of TI scout images by providing series of four adjacent scout images as input to their network. Yoo et al. [13] proposed the use of a localization network followed by a style-transfer network to obtain aligned BR-LGE scout images with a CINE-like contrast, followed by a convolutional neural network for blood-pool and myocardium segmentation. They ultimately performed automated TI selection by computing the mean signal intensities within the segmented region. While being fast and providing accurate results, the main drawback restricting the immediate and widespread adoption of deep-learning techniques into clinical routine remains their inline implementation. Existing solutions, such as incorporation of trained models into open-source image processing frameworks like Gadgetron [14], can be implemented. However, their installation is not straightforward and remains mainly used for research purposes at dedicated sites. To our knowledge, our algorithm was the first developed that enabled automated BL-LGE in clinical practice. The main findings of the study are listed below as follows:

1. Automated TI selection in BL-LGE can be quickly and accurately performed using a simple image-processing algorithm.
2. The proposed algorithm provides a highly reproducible TI selection with a variability with respect to manual selection lower than the inter- and intra-observer variability measured from two experts.
3. Automated TI selection can be easily implemented inline and used prior to a BL-LGE sequence to enable automated BL-LGE in clinical practice.

### Summary of the experiments and interpretation of the results

The automated TI algorithm has been tested on 120 retrospectively collected TI scouts consisting of 11 images each with TIs ranging from 60 to 160 ms in 10 ms increments. This number of images was a good tradeoff between acquisition speed (< 20 s) and the assurance of finding the optimal TI. However, the proposed algorithm can be used with any range and number of TIs. The optimal ROI size was derived from 40 patient scans. Rectangular ROI of size $\frac{{\mathrm{FOV}}_{\mathrm{shim}}}{2.5}$ and circular ROI of size $\frac{{\mathrm{FOV}}_{\mathrm{shim}}}{2.2}$ led to the same minimum absolute mean TI difference with manual expert. Only small differences were observed between rectangular and circular ROIs with dimension close to this optimum meaning that the algorithm can be robust to some variations in shim positioning around the provided guidelines. The main condition being that the ROI was entirely contained within the heart and encompassing both the myocardium and the blood-pool. On the 80 patients’ validation set, Fleiss kappa analysis revealed a good agreement between all TI selection methods. The automated-manual agreement was slightly higher than the intra-observer agreement and higher than the inter-observer agreement. The mean absolute difference and the percentage of matched optimal TIs confirmed this finding. The lowest mean absolute difference, respectively the highest percentage of matched TIs, was observed between the automated and manual selection, followed by the intra-observer and finally by the inter-observer selections. Moreover, under the exact same conditions, the automated algorithm, unlike manual selection, would enable perfect reproducibility in TI selection.

### Technical consideration, limitations, and future directions

During TI selection, two main problems can arise that require an additional acquisition. (1) The selection of a sub-optimal TI range, with a minimum TI too high or a maximum TI too low for optimal nulling of both healthy myocardium and blood-pools signals that can occur for an acquisition timing outside the expected time frame of 12–15 min post-gadolinium injection. (2) The presence of artifacts that could affect the TI selection process. Examples of TI scouts presenting these issues are visible in Figure Online Resource 6. Quality feedback is therefore needed to decide whether the whole-heart BL-LGE sequence can be performed with the selected TI or if a new scout acquisition is required. The problem of sub-optimal TI range can be easily addressed as it corresponds to the cases where either the first or the last image of the TI scout is selected. Acquiring new images using, respectively smaller or larger TIs until the selected TI is no longer the smallest or the largest value of the chosen range would address this issue. Examples of such cases and detection by the automated algorithm are visible in Figure Online Resource 7. The presence of artifacts in the ROI will result in a high number of hyperintense pixels and conversely in a smaller proportion of low intensity pixels. Artifact detection could be implemented by comparing the percentage of sub-thresholds pixels within the ROI of the selected image with the median percentage of sub-threshold values computed from a validation set. This quality control and re-acquisition remain to be implemented inline to enable robust automated BL-LGE.

The BL-LGE sequence employed in this study makes use of a T1ρ preparation module to generate a black-blood contrast, as recently validated by Sridi et al. [11] and Muscogiuri et al. [7]. The proposed TI selection technique could also be tested on different BL-LGE sequences such as T2-prepared [5] or magnetization transfer-prepared [8] sequences.

Finally, while providing good results, the robustness of the technique and especially the sensitivity of the method to deviation in shim positioning also need to be further quantified.

## Conclusion

We present a new algorithm for a reproducible and time efficient myocardial scar characterization using automated BL-LGE. Automated TI selection demonstrated to be fast and accurate with a performance slightly better than the manual variability observed between two experts. The algorithm’s simplicity enabled a straightforward on-site implementation while it’s explainability foster usage confidence and clinical acceptance. Moreover, the straightforward, yet efficient, design of the proposed automated algorithm provides several opportunities for other BL-LGE techniques. Inline testing in larger group of patients is now warranted.

### Supplementary Information

Below is the link to the electronic supplementary material.

Supplementary file1 (DOCX 3882 KB)

## Data Availability

Data and codes are available from the corresponding author on reasonable request.
