# Supplementary material for: Automated inversion time selection for black-blood late gadolinium enhancement cardiac imaging in clinical practice
Source: MAGMA. 2023 Jun 9;36(6):877–85. doi: 10.1007/s10334-023-01101-2 (PMC10667449; doi:10.1007/s10334-023-01101-2)
Supplement: Supplementary file 1 — Supplementary file1 (DOCX 3882 KB) [file 10334_2023_1101_MOESM1_ESM.docx]

**Supplementary Information**

**
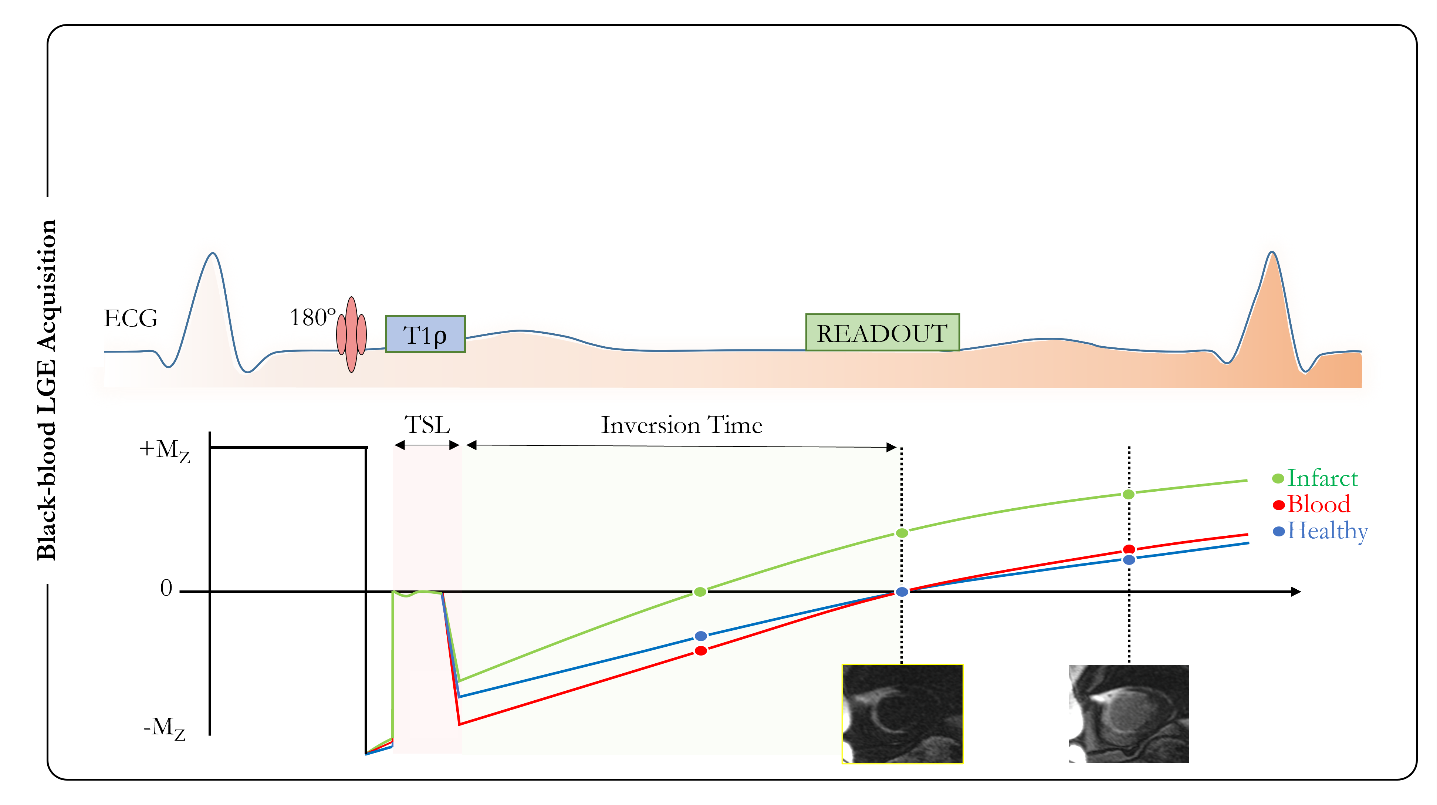
****Online Resource 1:** Schematic overview of the free-breathing single-shot 2D BL-LGE sequence. A 180 degrees inversion pulse is followed by an adiabatic T1ρ preparation module. The T1ρ preparation duration (TSL=27ms), controlling the degree of suppression of blood and viable myocardium signals, is fixed while the inversion time is selected prior to imaging with a dedicated scout scan. Abbreviations: BL, black-blood; LGE, late gadolinium enhancement; ECG, electrocardiogram; TSL, spin-lock time; IR, inversion recovery; M_0_ longitudinal magnetization; Acq, acquisition. This figure was duplicated from Sridi et al [11].

**Online Resource 2:** The location and dimension of the shim box are expressed in millimeters according to a three-dimensional Reference Coordinate System (RCS). The position of shim box in the Image Coordinate System (ICS), expressed in terms of pixels, can be computed using a mapping matrix as defined in Equation S1:

| $\left[ \begin{matrix} P_{x} \\ P_{y} \\ P_{z} \\ 1 \end{matrix} \right]=\left[ \begin{matrix} X_{x}\Delta_{i} & Y_{x}\Delta_{j} & 0 & S_{x} \\ X_{y}\Delta_{i} & Y_{y}\Delta_{j} & 0 & S_{y} \\ X_{z}\Delta i & Y_{z}\Delta_{j} & 0 & S_{z} \\ 0 & 0 & 0 & 1 \end{matrix} \right]\left[ \begin{matrix} i \\ j \\ 0 \\ 1 \end{matrix} \right]=M\left[ \begin{matrix} i \\ j \\ 0 \\ 1 \end{matrix} \right]$ | [S1] |
| --- | --- |

Where $P_{xyz}$ are the 3D coordinates of the voxel (i, j) in the RCS in mm. $S_{xyz}$ are the coordinates, in mm, of the first pixel of the image in the RCS, given by the Image Position Patient (Dicom attribute (0020,0037)). $X_{xyz}$ and $X_{xyz}$ are the values from the row (X) and column (Y) direction cosine of Image Orientation Patient (Dicom attribute (0020, 0032)), respectively. Variables $j$ and $i$ are the row and column indices to the image plane; the first column and row indices being zero.$\Delta_{j}$ and $\Delta_{i}$ are the row and column pixel resolution of Pixel Spacing in units of mm.

**
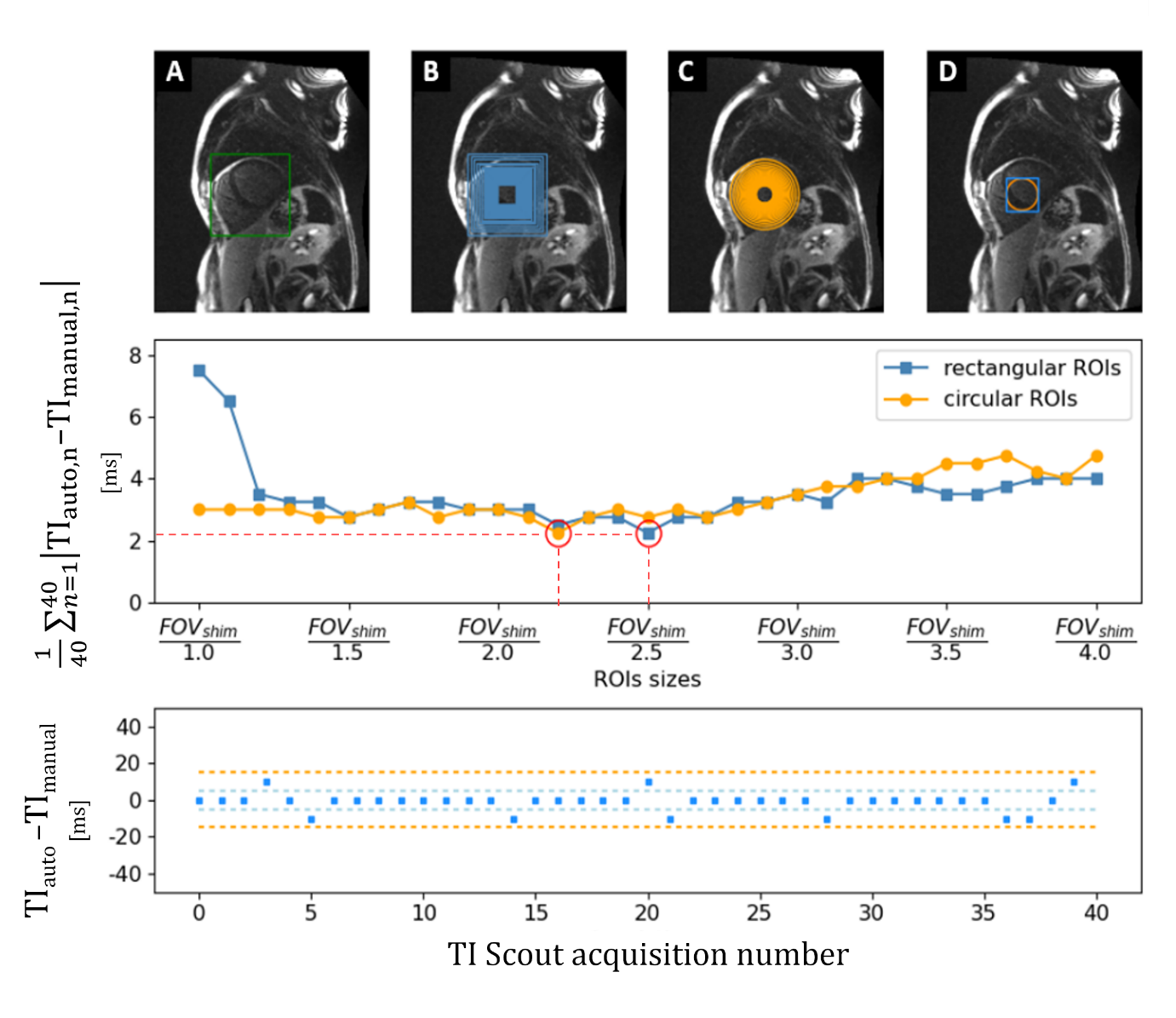
Online Resource 3:** ROI parameters optimization on 40 patients. Automated TI selection is performed for rectangular and circular ROIs with dimensions ranging from the size of the shim box to one fourth of its size with a decrement factor of 0.1. Mean absolute difference is computed between automated TI selection and an expert manual selection for each ROI. Top row: shim box (green), all rectangular ROIs (blue) and circular ROIs (orange) and the ROIs leading to the lowest mean absolute difference (MD) with respect to the manual selection for the two shapes. Middle row: MD between automated and manual TI selection over 40 subjects for all ROIs sizes. The size corresponding to the lowest MD are highlighted by red circles. Bottom row: Difference in index between the automated TI with the best rectangular ROI (size = $\frac{FOV_{shim}}{2.5}$) and the manual TI for the 40 different scouts.

|  | **Expert 1** | | **Expert 2** | |
| --- | --- | --- | --- | --- |
|  | **selection 1** | **selection 2** | **selection 1** | **selection 2** |
| **Automated algorithm** | 2.88 ± 4.53 | **1.88 ± 3.90** | **2.00 ± 4.00** | 3.12 ± 4.90 |
| **Expert 1** |  |  |  |  |
| **selection 1** | 0 | 2.75 ± 4.47^*^ | 3.62 ± 5.06^†^ | 3.75 ± 4.84^†^ |
| **selection 2** | - | 0 | 2.88 ± 4.79^†^ | 3.50 ± 5.02^†^ |
| **Expert 2** |  |  |  |  |
| **selection 1** | - | - | 0 | 2.62 ± 4.40^*^ |
| **selection 2** | - | - | - | 0 |
| *: Intra-expert; ^†^ Inter-expert | | | | |

**Online Resource 4:** Mean absolute difference in milliseconds between manual and algorithm-selected TI for 80 patient scouts. The mean absolute difference between automated TI and the experts’ different selections (2.47±4.38 ms) is lower than the mean intra-expert^*^ (2.69 ± 4.43 ms) and mean inter-expert^†^ (3.44±4.94 ms) variability.

**
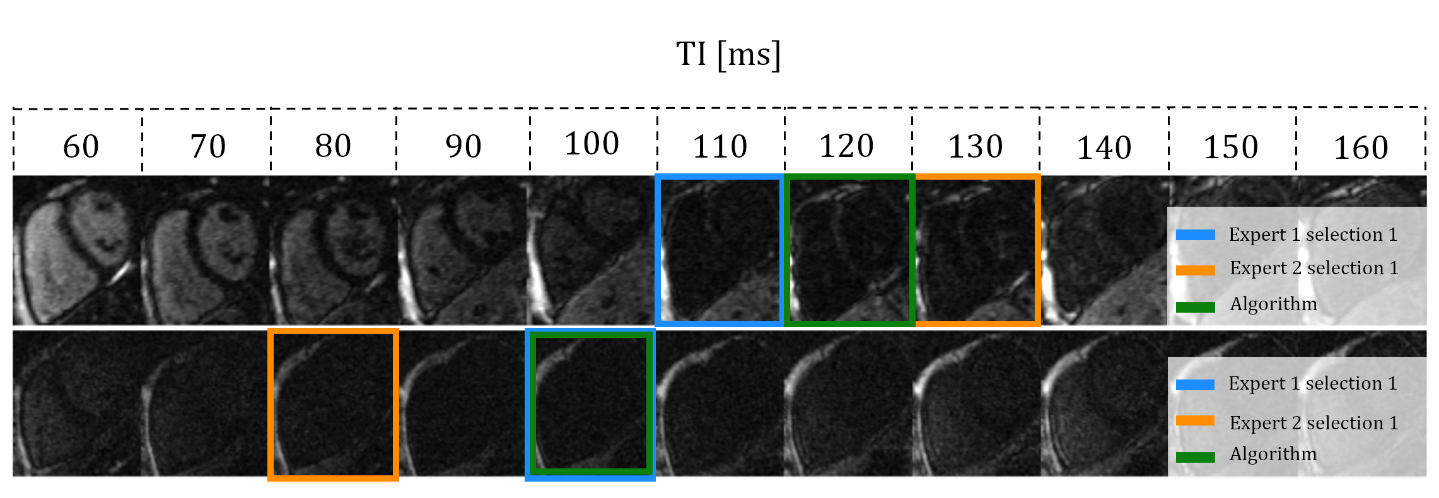
**

**Online Resource 5:** Visual inspection of the two TI scouts with differences in optimal selection higher than one image (i.e., TI difference >10ms). Top: Healthy myocardium and blood pool do not cancel at the same time. Bottom: adjacent images display very subtle contrast differences.

**
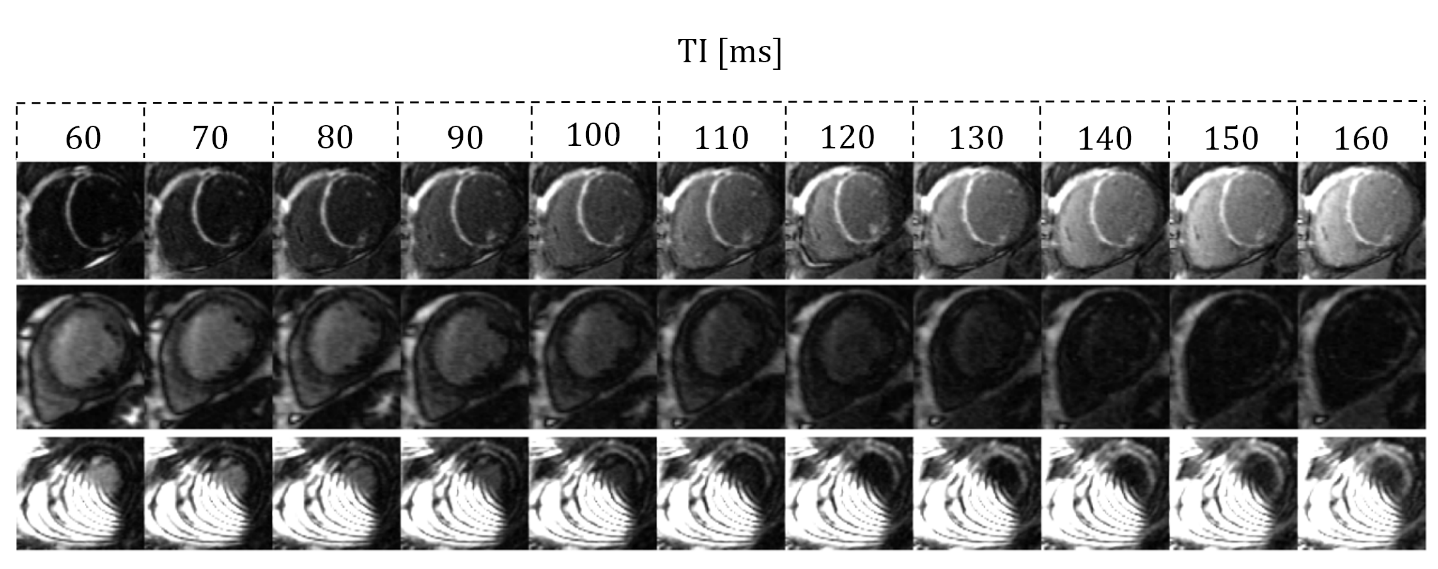
**

**Online Resource 6:** Examples of the main image quality issues that must be detected by the automated algorithm. Top row: minimum TI too high for optimal nulling of both healthy myocardium and blood pools signals. Middle row: maximum TI that is too low for optimal nulling of both healthy myocardium and blood pools signals Bottom row: presence of folding artifact.

**
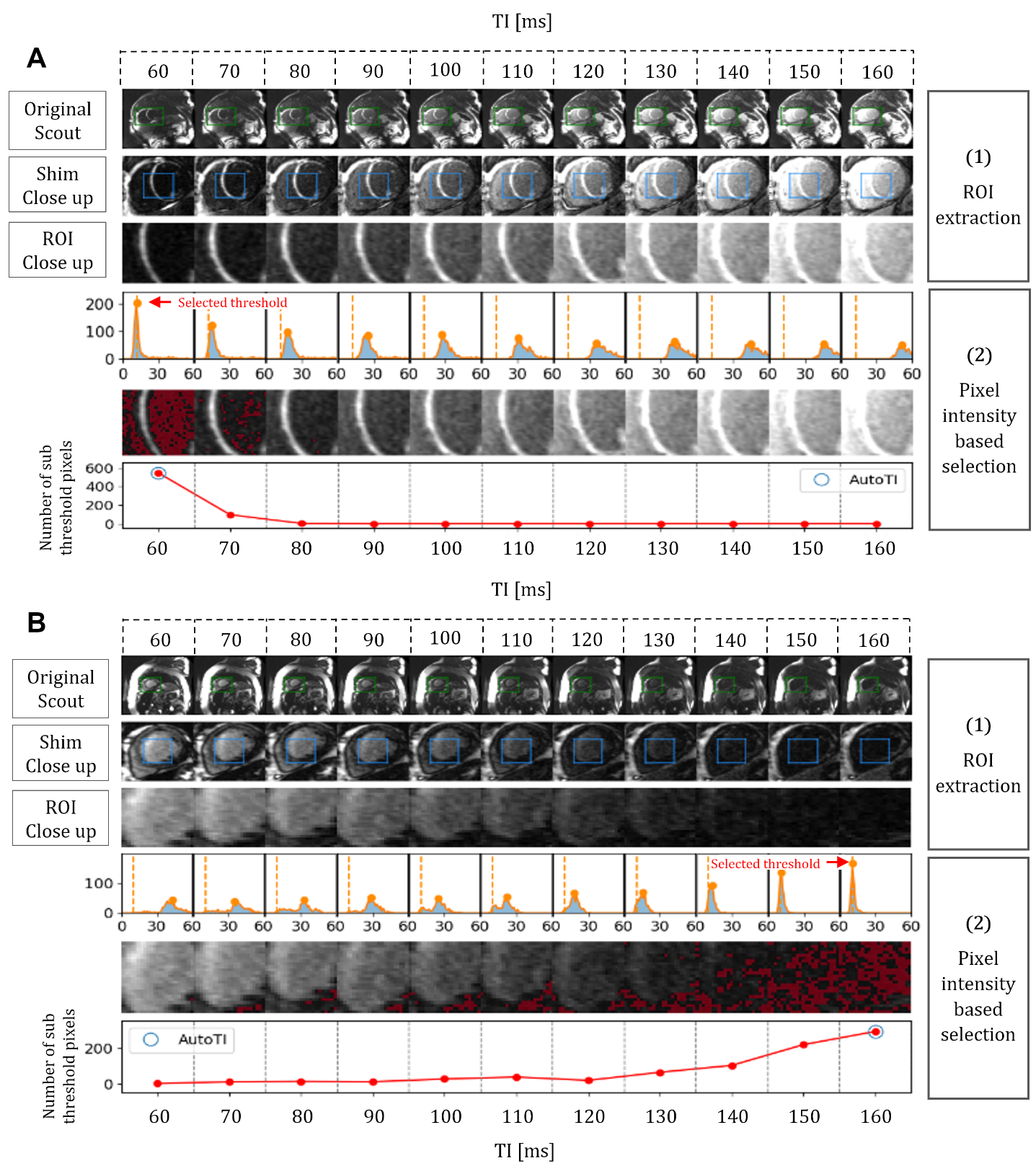
**

**Online Resource 7:** Quality feedback, examples of sub-optimal initial TI range detection. A) Minimum TI potentially too high for optimal nulling of both healthy myocardium and blood pools signal. The selected TI (blue circle) corresponds to the minimum range value (60ms). B) Maximum TI potentially too low for optimal nulling of both healthy myocardium and blood pools signal. The selected TI (blue circle) corresponds to the maximum range value (160ms).
